# Replication-Coupled Chromatin Remodeling: An Overview of Disassembly and Assembly of Chromatin during Replication

**DOI:** 10.3390/ijms22031113

**Published:** 2021-01-23

**Authors:** Céline Duc, Christophe Thiriet

**Affiliations:** UFIP UMR-CNRS 6286, Épigénétique et Dynamique de la Chromatine, Université de Nantes, 2 rue de la Houssinière, 44322 Nantes, France; celine.duc@univ-nantes.fr

**Keywords:** replication, chromatin, histones

## Abstract

The doubling of genomic DNA during the S-phase of the cell cycle involves the global remodeling of chromatin at replication forks. The present review focuses on the eviction of nucleosomes in front of the replication forks to facilitate the passage of replication machinery and the mechanism of replication-coupled chromatin assembly behind the replication forks. The recycling of parental histones as well as the nuclear import and the assembly of newly synthesized histones are also discussed with regard to the epigenetic inheritance.

## 1. Introduction

**Figure 1:**
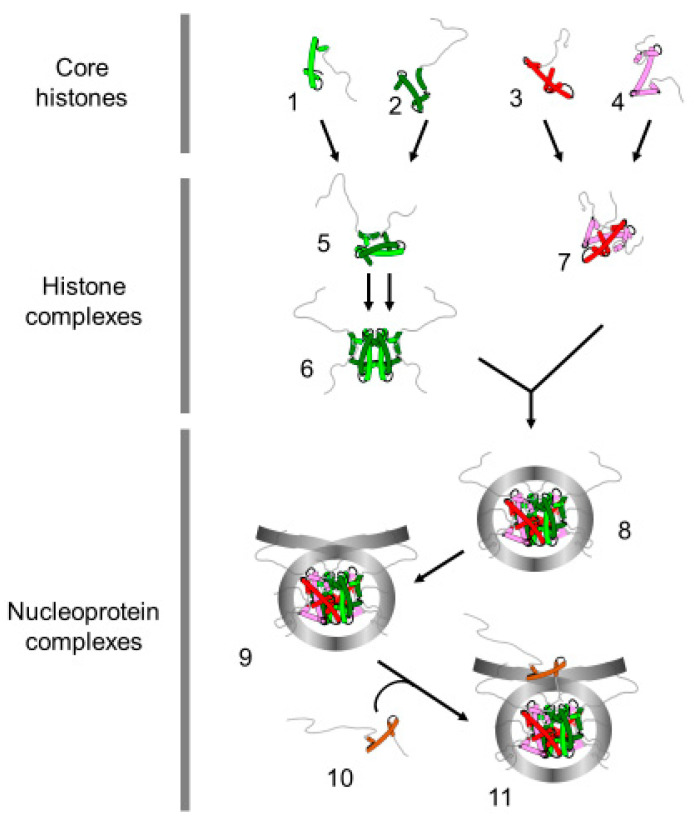
Terminology of the histone and nucleoprotein complexes. The drawing represents the proteins and nucleoprotein complexes that compose the basic unit of chromatin. H4 (1); H3 (2); H2B (3); H2A (4) (Core histones); H3/H4 dimer (5); (H3/H4) 2 tetramer (6), H2A/H2B dimer (7) (histone complexes); core particle (8) (146 bp of DNA); nucleosome (9) (>146 bp of DNA, presence of linker DNA); chromatosome (11) (nucleosome with linker histone (10)).

The doubling of genomic DNA occurring in the S-phase involves a timely regulated remodeling of the entire chromatin. Indeed, each replication site requires the displacement of nucleosomes in front of the fork to enable the progression of the replication machinery and the reformation of chromatin behind the replication fork [1,2]. The mechanism of histone eviction to facilitate the progression of the replication and replication-coupled chromatin assembly with parental and newly synthesized histones is conserved through the entire eukaryotic kingdom [2]. The histones are the most abundant proteins in the nucleus. It is generally believed that they have a dual function in eukaryotes, which consists of DNA packaging and regulation of DNA accessibility [3]. The histones are divided into five distinct major subtypes: the core histones, H2A, H2B, H3 and H4, and the linker histone, H1 [3,4]. In chromatin, the core histones form a tripartite octamer composed of two of each H2A, H2B, H3 and H4, wherein H3 and H4 are the central tetramers flanked by two dimers of H2A and H2B and wrapped by ~146 base pairs, forming therefore the nucleosome [5,6]. The fifth histone subtype is associated with extra DNA at the outer face of the nucleosome to generate the chromatosome [4,7] (Figure 1). The linker histone contributes to the higher-order structure of chromatin within the nucleus [8,9,10].

The formation of nucleoprotein structures between histones and DNA facilitates the organization of the eukaryotic genome within the nucleus. Moreover, the histones and, specifically, the amino-terminal tails of core histones are subjected to post-translational modifications, which are involved in genetic regulation [11,12]. Hence, it has been proposed that the variety of core histone modifications might constitute epigenetic signatures that continuously remodel chromatin for generating the molecular environment that enhances or impedes the accessibility of cellular machineries to DNA [13,14]. Three main types of actors are involved in the dynamics of these modifications [15]. Their deposition is performed by enzymes: the writers. When the histone is modified, the added mark is involved in the recruitment of specific chromatin factors: the readers. While the epigenetic modifications of histones contribute to chromatin dynamics and remodeling, these modifications are transient and require enzymes for their removal: the erasers. Even though the core histone tail modifications directly affect the chromatin activities, most modifications are not exclusive and require a combinatorial set of post-translational modifications for revealing biological functions, which led to the proposal that the post-translational modifications of core histones might constitute an epigenetic code [13,16]. Thus, the core histone tail domains are critical regulators of chromatin activities.

Importantly, during the S-phase of the cell cycle, the replication implies the doubling of the amount of DNA within the nucleus [17]. Hence, to preserve the compaction of the genetic information, novel histones are required. While DNA replication occurs exclusively within the nucleus, histone synthesis involves several steps, such as the transcription of the histone genes, the cytoplasmic translation of histone RNAs, the nuclear import of neo-synthesized histones and their assembly within chromatin. The nucleosome requires the four core histone proteins; the histone sequences present variations [18,19,20,21]. These isoforms are associated with specific chromatin processes such as transcription control, DNA repair or regulation of centromeric heterochromatin [20,21,22,23,24]. In addition to the variations in sequences, the histone variants differ from the canonical histone in their timing of synthesis [25,26]. Indeed, canonical core histone syntheses are controlled by the cell cycle and are produced only during the S-phase, which led to their definition as replication-dependent histones. In contrast, the core histone variants are synthesized throughout the interphase and are defined as replication-independent histones.

The complex mechanism of chromatin duplication has been extensively studied over the past few decades. Chromatin replication involves the remodeling of the entire genome once per cell cycle to facilitate the passage of replication machinery and the reassembly of nucleosomes behind the replication fork. The focus of this review is to summarize and discuss the current knowledge on the processes occurring in front of and behind the replication fork. Here, we emphasize the histone processing rather than the bevy of chaperones and factors involved (see for review [27,28,29,30]).

## 2. Chromatin Remodeling in Front of the Replication Fork

Chromatin replication is a timely regulated process, wherein the chromatin structure presents a hindrance to the cellular machinery. Thus, it is first required to withdraw the higher-order chromatin structure. The chromatin structure is relaxed by the removal of the linker histone, which is believed to affect chromatin folding within the nucleus. Indeed, analyses of the function of H1 in *Tetrahymena thermophila*, *Physarum polycephalum* and *Arabidopsis* revealed that the absence of linker histone significantly increased the nuclear volume [31,32,33]. Furthermore, in *Tetrahymena* and *Physarum*, it has been shown that phosphorylation of the linker histone recapitulates the absence of H1, suggesting that histone linker phosphorylation facilitates its removal from chromatin [32,34]. Interestingly, the natural synchrony of millions of nuclei within *Physarum* macroplasmodia has helped to show that knocking down the linker histone by siRNAs or the inhibition of phosphatase for increasing H1 phosphorylation resulted in the loss of the timing of replication [32]. Therefore, these results suggested that the release of the linker histone facilitates the reduction of chromatin packaging, which is possibly timely regulated during the S-phase in vivo. Besides the evidence of the functions of H1 histones in replication, it has been shown that linker histones exhibit critical roles in metazoan development by affecting the chromatin structure and the 3D organization within the nucleus [35,36,37].

**Figure 2:**
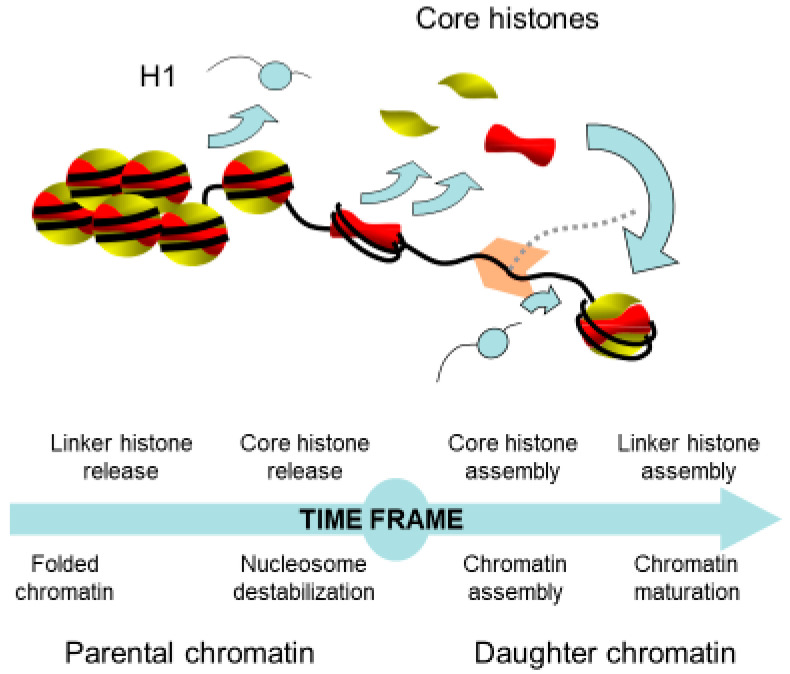
Time frame of the chromatin remodeling coupled to replication. The access of the replication machinery to DNA requires the well-controlled destabilization of chromatin in front of the replication fork and the reformation of chromatin onto the daughter strands. This latter process involves the recycling of parental and newly synthesized histones. Newly synthesized histones are necessary to compensate the deficit of histones due to the doubling of DNA.

In addition to the chromatin high-order structure disruption by the removal of the linker histone, it has been suggested that the nucleosomal structure impedes the passage of the replication machinery [38,39]. Therefore, core histones in front of the replication fork need to be released to allow DNA replication. In this respect, two models have been proposed: (i) either the nucleosome is disrupted with first the sequential removal of H2A/H2B dimers and of the H3/H4 tetramer; (ii) or the histone octamer is displaced. Indirect evidence using psoralen crosslinking revealed that, in front of the replication fork, there is a bubble of denatured DNA corresponding to the length of DNA protected by the H3/H4 tetramer [40]. This assay relies on the ability of psoralen to crosslink both strands of protein-free DNA, followed by electronic microscopy observations of the deproteinized DNA molecules under denaturing conditions. This protocol results in the visualization of single-stranded DNA bubbles corresponding to protein-protected DNA that are not cross-linked and of stretches of cross-linked double-stranded DNA that reflect regions free of proteins. If the lengths of DNA fragments can efficiently be measured, the observations of deproteinized DNA molecules do not definitely ensure the identity of proteins associated to DNA [41]. Notwithstanding the above, these experiments clearly showed that the length of destabilized chromatin in front of the replication fork is less than 1 kb [39,42] (Figure 2). The dissolution of the nucleosome in front of the replication fork raises the question of the fate of the parental histones. Indeed, one can conceive that these proteins are recycled or degraded. This concern has been addressed by different experimental approaches, which demonstrated that replicated chromatin is composed of one half of recycled parental histones and the other half of newly synthesized histones [43,44,45,46,47]. Notably, the transfer of parental histones as well as the transportation of new histones require chaperones in vivo for neutralizing the negative charges of histones and to prevent their binding to RNA, as histones bind RNA with an approximate 100-fold greater affinity relative to DNA at physiological ionic strength and, therefore, are never free in the cell but always escorted by chaperones [27,30,48].

## 3. Nuclear Transportation of New Histones

New histone proteins are required for restoring the nucleosome density behind the replication fork and are translated into the cytoplasm [38]. This raises the question of how histone proteins are transferred into the nucleus and which players are required for achieving this process. Although canonical histones are highly conserved proteins in eukaryotes, the genomic organization of their genes presents striking differences between animal and plant kingdoms [25,49]. In plants, the few copies of the histone genes are dispersed within the genome and the histone mRNAs are polyadenylated [50]. In contrast, in animals, the multiple histone genes are clustered within the genome and their transcripts are devoid of polyadenylation [25,51]. Despite these differences, in both kingdoms, the histone genes are individually transcribed and individual histones are therefore produced. The mechanism by which core histones are transferred into the nucleus is still controversial. Indeed, experiments using HeLa cells for examining the nuclear transportation of H3 and H4 exhibited opposite results whereby the histones were transported into the nucleus as monomers or as dimer of H3/H4 [52,53,54,55]. Whether these data reflect the putative two cellular mechanisms of nuclear import of histones or discrepancies in the experimental approaches remain unclear. However, the data from TAP-Tag experiments strongly suggested that cytoplasmic histones associate with four distinct chaperone complexes [52]. Using the protozoan, *Physarum polycephalum*, which presents a large population of naturally synchronous nuclei and the unique ability to spontaneously incorporate exogenous proteins, we have shown that the incorporation of trace amounts of histone monomers in the S-phase was not retrieved in the nuclear fraction, while the incorporation of a similar amount of the H3/H4 histone complex at the same cell cycle stage was rapidly transported into the nucleus [55]. These results strongly suggested that, at least in *Physarum*, the efficient nuclear import of H3 and H4 during the S-phase required the formation of the H3/H4 complex within the cytoplasm, meaning that the histones are correctly folded when transported within the nucleus.

Pioneer analyses of newly synthesized histones revealed that cytoplasmic H4 is acetylated in dividing avian erythroblasts, suggesting that, shortly following their synthesis, the histone is enzymatically modified [56]. These results were later confirmed in other organisms including humans and the lysine acetylations are localized at positions 5 and 12, except in *Tetrahymena*, which lacks the arginine 3; therefore, lysine acetylations are shifted at positions 4 and 11 [57,58]. The cytoplasmic acetylation of H4 was reinforced by the purification of the HAT-B from *S. cerevisiae* cytoplasmic extracts, which exclusively acetylates non-nucleosomal lysine 12 of H4 [59,60,61]. Altogether, these data suggested that the histone amino-terminal tails have a function in the nuclear import of newly synthesized histones during the S-phase of the cell cycle [62]. Investigations using genetic approaches in budding yeast revealed that the amino-terminal tail of H4 and of H3 is important for the maintenance of chromatin structure over the generations but were not able to conclude on the role of these histone domains in nuclear import [63]. Insights into the function of the amino-terminal tail of histones in the nuclear import have been provided by experiments on the incorporation of exogenous histones in the slime mold *Physarum polycephalum* during the S-phase [55,61]. In these experiments, negligible amounts of tagged extracellular histones are spontaneously incorporated into the cytoplasm, which presented no detectable effects on cellular homeostasis and cell cycle progression [64,65]. The nuclear import of the exogenous histones was determined by examining their accumulation into the nuclear fraction. Nuclear import was strongly inhibited when examining exogenous complexes including the H3/H4 complex lacking the H4 amino terminal tail, showing that this region of H4 is critical for the transportation of the H3/H4 histone complex within the nucleus [61]. Further experiments on the ectopically positioned amino-terminal tail of H3 and of H4 revealed that the H4 amino domain is not sufficient for the nuclear import of the H3/H4 complex [55]. Indeed, swapping the amino termini of H3 and H4 almost completely abolished the nuclear distribution of the complex, revealing that the histone amino termini and the fold domains act synergistically in nuclear import. Although the role of H2A and H2B amino termini in nuclear import has been examined less extensively than that of H3 and H4, the experiments in *Physarum* revealed that their absence has no effect on the nuclear localization of the exogenous H2A/H2B complexes [66]. These results suggested distinct mechanisms of nuclear import of H2A/H2B dimer and H3/H4 complex that involve different histone chaperones (see for review [67]).

## 4. Assembly of Nucleosome behind the Replication Fork

**Figure 3:**
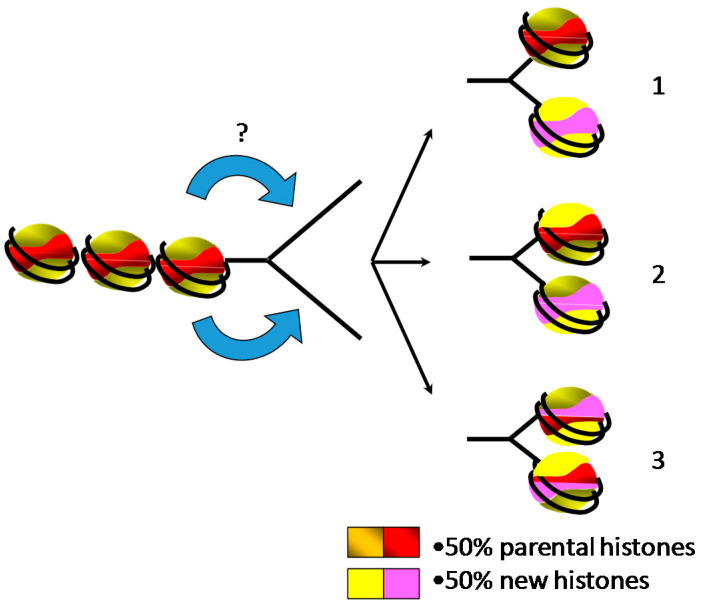
Two halves of parental and newly synthesized histones are assembled behind the replication fork onto the daughter strands for restoring the parental chromatin landscape, leading to three putative models of distribution. (**1**) Parental core histones and newly synthesized histones form distinct octamers. (**2**) Parental and new histone complexes (H2A/H2B dimers and H3/H4 tetramers) are mixed during the assembly. (**3**) The smallest stable complexes of parental and new histones (H2A/H2B dimers and H3/H4 dimers) are randomly mixed for forming core histone octamers.

Over the past few decades, replication-coupled assembly of chromatin has been extensively studied to understand how the cell packages the genetic material after its doubling [68]. Although details about the formation of chromatin behind the replication forks are regularly revisited, the proposed models are still under debate (Figure 3). Nevertheless, several lines of evidence demonstrated that following replication, chromatin is composed by one half of parental histones and the other half of newly synthesized histones [46]. One of the first concerns was to determine the pattern of distribution of the new and the parental histones on the leading and the lagging strands. Indeed, two distinct models can be drawn: the parental histones are transferred onto one strand of replicated DNA and the new histones are assembled onto the other strand, or parental and new histones are randomly distributed onto both strands of DNA [69,70]. Historical experiments were carried out in the presence of cycloheximide to prevent the synthesis of new histones followed by the investigation of the nucleosome distribution at the replication forks [38,44]. However, results obtained from different analyses were controversial. Electron microscopy observations revealed the presence of stretches of DNA depleted of “beads”, suggesting a conservative segregation of the parental nucleosomes [45]. In contrast, biochemical analyses using micrococcal nuclease exhibited a decrease in nucleosome density due to the absence of new histones but their distribution was found on both arms of neo-synthesized DNA, suggesting a dispersive distribution of the parental nucleosomes behind the replication fork [71]. These discrepancies are most likely related to the experimental conditions wherein the preparation of the electronic microscopy samples might favor the eviction of the less stable nucleosomes within a single molecule, while the biochemical analyses might result in the mixture of different populations within the sample. Furthermore, it is doubtful whether the use of cycloheximide to prevent the synthesis of new histones has no additional side effects. Recently, NGS (Next Generation Sequencing) analyses of sister chromatids using post-translational modifications of H4 (H4K20me2 for parental histones and H4K5ac for new histones) have shown that both old and new histones are distributed on both strands of DNA [72]. However, detailed analyses of the data revealed that the partitioning of parental histones is not perfect, with a slight bias in parental histone recycling to the leading and lagging strands, suggesting distinct mechanisms of chromatin assembly. Nonetheless, these experiments might be biased by the examination of histone modifications that have a distinct half-life in vivo and are used as a proxy for parental and new histones rather than examining the actual parental and new histones.

It is generally believed that chromatin assembly is a stepwise process wherein the H3/H4 tetramer is first loaded onto DNA, followed by the deposition of the two heterodimers of H2A/H2B [73]. This model has originated from biochemical experiments of the purification of cellular histones, in which it was shown that the increase in salt concentration released first linker histone H1, then H2A/H2B and finally H3/H4 [74]. Thus, it was proposed that the assembly of the nucleosome might follow the opposite stepwise mechanisms of nucleosome destabilization. Actually, in vitro salt dialysis nucleosome reconstitution supports this model as the nucleosome reconstitution consists in the mixture of DNA and core histones in 2M NaCl and the nucleosomal complex forms by the gradual decrease of NaCl concentration [75]. Consistently, the H3/H4 chaperone CAF-1 (Chromatin Assembly Factor-1) assembled in vitro H3/H4 tetramer onto chromatin concomitantly with replication in the absence of H2A/H2B [76]. Nonetheless, when the four core histones are present in the mixture, replication-coupled nucleosome assembly is promoted by CAF-1 [77]. Although in vitro analyses of replication-coupled nucleosome assembly using cell-free systems did not provide an unambiguous confirmation of the stepwise mechanism of assembly of nucleosome, analyses carried out in cellular systems tended to refute this sequential model [38]. Indeed, pioneer analyses showed that chromatin behind the replication fork exhibited an altered structure of newly replicated chromatin that affected nuclease digestion rates, but not the overall digestion patterns of DNase I and micrococcal nuclease or the core histone composition [78,79]. Most likely, the alterations of the chromatin conformation of newly replicated chromatin reflected the absence of the linker histone, as suggested by the size of the repeat subunit [79]. Furthermore, electronic microscopy observations of psoralen-crosslinked newly replicated chromatin failed to reveal at first small single-stranded DNA bubbles corresponding to H3/H4 tetramer bound to DNA within the large population that was examined [40]. Altogether, these in vivo data suggest that the sub-nucleosomal H3/H4 tetramer is not a requirement for nucleosome assembly, even though it has been proposed that this absence of detection might be due to the fast association of H2A/H2B to the sub-nucleosomal H3/H4 tetramer [40]. Although sequential deposition of core histone octamer onto newly replicated DNA remains elusive, it has been shown that new and old histone complexes can intermix [80]. Furthermore, it has been shown that the replisome (set of factors that coordinate efficient DNA replication) guides nucleosome assembly by interacting with histones and histone chaperones [81]. Subsequent to the assembly of core histones, it is generally believed that linker histone is added to form the chromatosome, contributing to chromatin maturation and the recovery of the parental chromatin structure that can be experimentally monitored by the decrease in nuclease accessibility [73]. The direct evidence of the role of histone H1 in chromatin organization within the nucleus and the control of the epigenetic landscape has been provided by chromosome conformation capture analyses and the alteration of epigenetic marks [37].

## 5. Inheritance of Epigenetic Marks during Replication

Even though the mechanism of the deposition of the histone octamer behind the replication fork remains unclear, in recent years, it became important to explore the mechanism of inheritance of the epigenetic marks. This has been investigated by several groups, who revealed a number of difficulties in its achievement. The synchronization of the cells is clearly a critical point, as with any experiments focused on replication studies. The analyses of the inheritance of epigenetic marks require also the discrimination of newly synthesized histones and parental ones, and neo-synthesized DNA and parental DNA. Different strategies have been exploited for studying the transmission of the histone modifications following replication, which do not ease the interpretation and comparison of the details [46,82]. Importantly, the inheritance of epigenetic mark following replication fork passage means that replication does not modify chromatin organization, and thus, nucleosome positioning should be almost identical between the parental chromatin and daughter ones. This assumption was verified by Schlissel and Rine in labeling in living yeast positioned nucleosomes and determined their fate following replication [83]. Clearly, the results of these experiments revealed the recovery of the labeled nucleosomes through DNA replication without local movement along the chromatin fiber. Even though the latest data showed the repositioning of parental nucleosomes behind replication forks in yeast, a similar approach developed in mouse embryonic stem cells demonstrated that the positional inheritance of parental nucleosomes is not verified throughout the genome [82]. Indeed, the authors observed the local redeposition of the parental nucleosome through DNA replication in repressed chromatin domains, while active chromatin domains exhibited the positional dispersion of parental nucleosomes in a replication-dependent manner. Interestingly, in addition to the structure of chromatin in the recycling of parental histones, in vitro analyses of real-time single-molecule in *Xenopus laevis* egg extracts revealed that the fate of the parental nucleosome did not follow a unique mechanism [84]. In these experimental conditions, the nucleosome in front of the replication fork exhibited different behaviors: sliding along the DNA fiber, stalling replication fork, histone transfer or histone eviction. Notably, depletion of H3/H4 in the egg extracts showed a decrease in the nucleosome eviction in favor of the nucleosome transfer, which is abolished when depleted egg extracts are supplemented with recombinant histones. These data strongly suggest that the amount of free histones controls the recycling of parental histones. Even though the role of free histones has not been demonstrated in replication in vivo, similar observations have been reported in transcription in living cells [85,86]. Thus, data resulting from experiments showing the overexpression of histones might affect to some extent the equilibrium between free and chromatinized histones. To prevent this putative artefact, experiments have been carried out using labeling of endogenous histones with non-radioactive isotopes for SILAC (Stable Isotope Labeling by Amino acids in Cell culture) analyses coupled to EdU (Ethyl deoxy Uridine, an analog of Thymidine) pulses for resolving neo-synthesized DNA. This strategy was able to show that histone modifications propagate in a distinct mode across the cell cycle [46]. 

While it is generally believed that histone modifications contribute to the epigenetic information, histone variants have been associated with chromatin activities and, like histone modifications, are part of the epigenetic information [87]. Therefore, the epigenetic inheritance requires histone variant repositioning behind the replication fork and synthesis of new variants to compensate the genome duplication. As an example of this inheritance of histone variants, parental H3.1 and H3.3 have been examined microscopically by SNAP-tag labeling [88]. Interestingly, these analyses revealed that parental H3.3 and H3.1 display distinct patterns of distribution through the S-phase related to the timing of replication. Indeed, the authors report that H3.3 is preferentially associated with early replicating regions, and conversely, H3.1 marks late replicating regions. These results suggest that regions with parental histones H3.1 and H3.3 contribute to replication timing control. Very little is known about H3.3 in the S-phase and its role in the epigenetic inheritance. However, this histone variant has been defined as the replacement isoform associated with transcription, suggesting that transcription and replication present interdependencies during the cell cycle. The coupling between transcription and replication was suggested in the early 1980s in the slime mold *Physarum polycephalum* [89]. More recently, it has been shown that factors involved in transcription compete with the nucleosome for associating with neo-synthesized DNA and transcription is involved in the positioning of the nucleosome following replication [90,91].

## 6. Concluding Remarks

In the present overview of chromatin remodeling associated with replication, we pinpointed some concerns that have been the focus of extensive research over the past few decades. Understanding the fine details of how the chromatin states are transmitted from one generation to the next one has a broad range of applications in life sciences. This leads researchers to revisit basic questions of chromatin replication for improving our knowledge, even though the basic chromatin subunit assembled behind the replication fork is a mixture of new and parental histones, which remains elusive. With the increasing evidence of relationships between epigenetics and cell destiny, recent works have hinted at the inheritance of histone modifications of parental histones through replication. It would be also interesting to collect experimental data on the inheritance of the parental histone variants and determine whether the positioning of new histone variants synthesized in the S-phase results in chromatin rearrangement behind replication fork. We must keep in mind that besides the obvious dynamics of replication, the chromatin subunit is not static as histone complexes are susceptible to exchange at different rates in living cells. The perspective of experiments providing information on the kinematics of chromatin replication in the nucleus rather than snapshots offers an exciting glimpse into the mechanisms of eukaryotic genetic transmission. It is certain that the field of chromatin biosynthesis will remain a challenging topic for future investigations.
